# β1 integrin is essential for blood–brain barrier integrity under stable and vascular remodelling conditions; effects differ with age

**DOI:** 10.1186/s12987-023-00453-0

**Published:** 2023-07-03

**Authors:** Sebok K. Halder, Violaine D. Delorme-Walker, Richard Milner

**Affiliations:** San Diego Biomedical Research Institute, 3525 John Hopkins Court, Suite 200, San Diego, CA 92121 USA

**Keywords:** Brain, Aging, Blood vessels, β1 integrin, Chronic mild hypoxia, Blood–brain barrier integrity

## Abstract

**Background:**

Maintaining a tight blood–brain barrier (BBB) is an important prerequisite for the preservation of neurological health, though current evidence suggests it declines with age. While extracellular matrix-integrin interactions play critical roles in regulating the balance between vascular stability and remodeling, it remains to be established whether manipulation of integrin function weakens or strengthens vascular integrity. Indeed, recent reports have generated conflicting outcomes in this regard.

**Methods:**

Here, in young (8–10 weeks) and aged (20 months) mice, we examined the impact of intraperitoneal injection of a function-blocking β1 integrin antibody, both under normoxic conditions, when the BBB is stable, and during chronic mild hypoxic (CMH; 8% O_2_) conditions, when a vigorous vascular remodeling response is ongoing. Brain tissue was examined by immunofluorescence (IF) for markers of vascular remodeling and BBB disruption, and microglial activation and proliferation. Data were analyzed using one-way analysis of variance (ANOVA) followed by Tukey’s multiple comparison post-hoc test.

**Results:**

In both young and aged mice, β1 integrin block greatly amplified hypoxia-induced vascular disruption, though it was much less under normoxic conditions. Interestingly, under both normoxic and hypoxic conditions, β1 integrin antibody-induced BBB disruption was greater in young mice. Enhanced BBB breakdown was associated with increased levels of the leaky BBB marker MECA-32 and with greater loss of endothelial tight junction proteins and the adherens protein VE-cadherin. Surprisingly, β1 integrin blockade did not reduce hypoxia-induced endothelial proliferation, nor did it prevent the hypoxia-associated increase in vascularity. Commensurate with the increased vascular disruption, β1 integrin blockade enhanced microglial activation both in young and aged brain, though the impact was much greater in young brain. In vitro studies revealed that β1 integrin blockade also reduced the integrity of a brain endothelial monolayer and triggered disruptions in tight junction proteins.

**Conclusions:**

These data demonstrate that β1 integrin plays an essential role in maintaining BBB integrity, both under stable normoxic conditions and during hypoxia-induced vascular remodeling. As β1 integrin blockade had a greater disruptive effect in young brain, effectively shifting the BBB phenotype of young brain towards that of the aged, we speculate that enhancing β1 integrin function at the aged BBB may hold therapeutic potential by reverting the deteriorating BBB phenotype back towards that of the young.

**Supplementary Information:**

The online version contains supplementary material available at 10.1186/s12987-023-00453-0.

## Introduction

By having the specialized properties of high electrical resistance and low permeability, blood vessels of the central nervous system (CNS) are uniquely well adapted to protect vulnerable neural cells from potentially harmful components in the blood [1, 2], as well as allowing selective transport of only those metabolites (e.g.: glucose and amino acids) required by CNS resident cells. In this manner, CNS blood vessels constitute a very selective barrier, referred to as the blood–brain barrier (BBB) that effectively separates the blood and CNS compartments. The molecular basis of the BBB lies in a combination of structures, including endothelial adherens and tight junction protein complexes, extracellular matrix (ECM) proteins of the vascular basement membrane (BM), and the influence of adjacent CNS-resident cells such as astrocytes, pericytes, and microglia [3–6]. BBB disruption is a central pathogenic component of many neurological diseases, including meningitis, ischemic stroke, multiple sclerosis (MS), and CNS tumors [7–9]. Importantly, current evidence suggest that BBB integrity is also compromised with advancing age [10, 11], and that sustained insults to the BBB may predispose to the development of vascular dementia by disrupting the normal homeostatic equilibrium present within the CNS milieu, leading to neuronal dysfunction and neurodegeneration [12, 13].

ECM proteins of the vascular BM include laminin, collagen IV, fibronectin and perlecan, and play important instructive roles in regulating vascular cell behavior, not only during development and vessel maturation, but also in adult tissue, both during physiological and pathological vascular remodeling [14–16]. The ECM protein fibronectin is expressed at high levels during developmental brain angiogenesis and is re-expressed at high levels during vascular remodeling in the adult, where it plays a key role in driving endothelial proliferation and migration [17–20]. In contrast, other ECM proteins such as laminin and collagen IV appear later in development [19] and appear to promote endothelial differentiation and vascular stability [21, 22]. ECM proteins mediate their effects via cell surface heterodimeric receptors called integrins, of which the β1 class is the most abundant [23]. Consistent with these developmental changes in ECM proteins, we previously demonstrated that during brain development, blood vessels switch from expressing high levels of fibronectin and its cognate receptor α5β1 integrin early on, to high levels of laminin and its cognate receptor α6β1 integrin at later timepoints [19]. To further understand the contribution of β1 integrin function to vascular integrity, several studies have examined the impact of function-blocking reagents or genetic deletion. Intriguingly, these studies have generated conflicting results, with some showing that β1 integrin blockade stabilizes vascular integrity, both in the brain and other organs [9, 24, 25], while others showed the opposite result [26–28].

When mice are exposed to chronic mild hypoxia (CMH; 8% O_2_), this triggers a strong vascular remodeling response in the CNS, resulting in 50% increased vessel density over two weeks which is also associated with transient disruption of BBB integrity [29, 30]. As current evidence suggests that BBB integrity declines with age [10, 11], we recently compared the hypoxia-induced cerebrovascular remodelling response in young (8–10 weeks) and aged (20 months) mice. This demonstrated that aged mice show delayed vascular remodelling that is associated with greatly increased BBB breakdown [31]. Here we aimed to investigate this link further by asking the following questions: (i) is the increased vascular leak and delayed vascular remodeling of aged brain due to attenuation of the angiogenic fibronectin-α5β1 integrin signalling axis, and (ii) does pharmacological blockade of β1 integrins prevent vascular remodeling and thereby stabilize BBB integrity, or alternatively, does it disrupt endothelial-ECM interactions, thereby enhancing BBB breakdown?

## Materials and methods

### Animals

The studies described were reviewed and approved by the Institutional Animal Care and Use Committee at San Diego Biomedical Research Institute (SDBRI). Young and aged female C57BL6/J mice were obtained from Jackson Laboratories and the NIH National Institute on Aging rodent colony and were maintained under pathogen-free conditions in the closed breeding colony of SDBRI.

### Chronic hypoxia model

Female C57BL6/J mice, 8–10 weeks (young) and 20 months (aged), were housed 4 to a cage, and placed into a hypoxic chamber (Biospherix, Redfield, NY) maintained at 8% O_2_ for periods up to 14 days. Littermate control mice were kept in the same room under similar conditions except that they were kept at ambient sea-level oxygen levels (normoxia, approximately 21% O_2_ at sea-level) for the duration of the experiment. Every few days, the chamber was briefly opened for cage cleaning and food and water replacement as needed.

### Administration of β1 integrin blocking antibody

Mice received daily intraperitoneal (i.p.) injections of either the anti-mouse β1 integrin function-blocking antibody (clone HMβ1-1) or an isotype control antibody (clone Ha4/8) both at doses of 2.5 mg/kg (BD Bioscience, La Jolla, CA, USA).

### Immunohistochemistry and antibodies

Immunohistochemistry was performed on 10 µm frozen sections of cold phosphate buffer saline (PBS) perfused tissues as described previously [32]. Monoclonal antibodies from BD Biosciences reactive for the following antigens were used in this study: CD31 (clone MEC13.3; 1:300), MECA-32 (1: 100), Mac-1 (clone M1/70; 1:50), CD68 (clone FA-11; 1:2000), and the integrin subunits α1 (clone Ha31/8; 1:100), α5 (clone MFR5; 1:100), α6 (clone GoH3; 1:500) and β1 (clone 9EG7; 1:100). The hamster anti-CD31 (clone 2H8; 1:500) monoclonal was obtained from Abcam (Cambridge, MA, USA). Rabbit antibodies reactive for the following proteins were also used: Ki67 (1:4000 from Novus Biologicals, Centennial, CO), fibronectin (1:1500 from Sigma, St. Louis, MO), laminin (1: 2000 from Sigma), fibrinogen (1:1500 from Millipore, Temecula, CA, USA), and claudin-5 (1:3000) and ZO-1 (1:1500) both from Invitrogen, Carlsbad, CA, USA. Goat anti VE-cadherin antibody (1:300) was obtained from R&D Systems. Sheep anti-fibrinogen antibody (1:3000) was obtained from Bio-Rad. Secondary antibodies used (all at 1:500) included Cy3-conjugated anti-rabbit, anti-rat, anti-hamster, and anti-sheep, Cy5-conjugated anti-rabbit from Jackson Immunoresearch, (West Grove, PA, USA) and Alexa Fluor 488-conjugated anti-rat and anti-hamster from Invitrogen (Carlsbad, CA, USA).

### Image analysis

Images were taken using a 5X, 10X or 20X objective on an Axioskop2 plus microscope (Carl Zeiss, Dublin, CA, USA) equipped with an Infinity 3S camera (Lumenera, Ottawa, ON, Canada) and Infinity Analyze imaging software (Lumenera). For each antigen in all analyses, images of at least three randomly selected areas were taken at 5X, 10X or 20X magnification per tissue section and three sections per brain analyzed to calculate the mean for each animal (n = 4–9 mice per group). For each antigen in each experiment, exposure time was set to convey the maximum amount of information without saturating the image and was maintained constant for each antigen across the different experimental groups. The number of vascular leaks or MECA-32 + vessels per field of view (FOV) was quantified by capturing images and performing manual counts of the number of vessels showing extravascular leaked fibrinogen or MECA-32, respectively. The number of activated microglia was quantified by performing manual counts of the number of CD68 + cells or by morphological criteria of Mac-1 staining (large cell body and short process extensions) per FOV. Total Mac-1 area fluorescent signal per FOV was measured and analyzed using NIH Image J software. Endothelial and microglial proliferation was quantified by counting the number of CD31/Ki67 or Mac-1/Ki67 dual-positive cells per FOV, respectively. The number of vessels lacking expression of the tight junction proteins ZO-1 and claudin-5 was quantified by capturing images and performing manual counts. Each experiment was performed with 4–9 different animals per condition, and the results expressed as the mean ± SEM. Statistical significance was assessed using one-way analysis of variance (ANOVA) followed by Tukey’s multiple comparison post-hoc test or Student’s t test, in which p < 0.05 was defined as statistically significant.

### Cell culture

The immortalized bEnd3 brain endothelial cell line was obtained from the American Tissue Culture Collection (ATCC; Manassas, Virginia). bEnd3 cells were cultured in high glucose DMEM (Gibco) supplemented with 10 mM Glutamine (Invitrogen), 100 U/mL penicillin/100 U/mL streptomycin (Invitrogen), and 10% FBS (Millipore). Cells were maintained at 37 °C in a humidified atmosphere (5% CO_2_/95% air). Medium was changed every 3–4 days until cells reached confluence.

### Measurement of paracellular permeability

Effects of the anti-β1 antibody (clone HMβ1-1) on endothelial monolayer integrity were assessed by measuring paracellular permeability to three labeled dextrans: 4 kDa FITC-conjugated dextran (Milllipore-Sigma), 10 kDa Cascade Blue-conjugated dextran (Invitrogen) and 40 kDa Texas Red-conjugated dextran (Invitrogen). Briefly, transwell inserts (clear polyester (PET) membranes, 6.5 mm diameter, 0.4 μm pores, Cat. #3470, Corning) were coated with 30 µg/ml Laminin-111 (Milllipore-Sigma) for 2 h at 37 °C. bEnd3 cells were seeded on the apical side at a density of 5 × 10^4^ cells/cm^2^. Two hours after seeding, the isotype control antibody (clone Ha4/8, BD Pharmingen) or anti-β1 antibody (clone HMβ1-1) both at 10 µg/ml were added to the cell media. Each condition was performed in duplicate. Function-blocking β1 antibody and control isotype were refreshed every 24 h. Endothelial monolayer permeability was measured 48 h later. bEnd3 containing inserts were washed with Hank’s balanced salt solution (HBSS) + 10 mM HEPES pH 7.5 before being incubated with 700 μl HBSS + 10 mM HEPES pH 7.5 in the basolateral side and 300 μl HBSS + 10 mM Hepes pH 7.5 containing 10 µg/ml of each fluorescently labeled dextran on the apical side. Inserts were incubated with tracers for 1 h at 37 °C. The medium from the basolateral well was collected and fluorescence intensity was measured in triplicate on a VersaMax Spectrophotometer using the following wavelengths: Ex405/Em440 for the Cascade Blue-conjugated dextran, Ex485/Em525 for the FITC-conjugated dextran and Ex560/Em615 for the Texas Red-conjugated dextran. The concentrations of the corresponding dextrans were determined and the permeability coefficient values were calculated using the following equation:

$${\text{P }} = \, \left( {{\text{Vr}}/{\text{C}}0} \right) \, \times \, \left( {{1}/{\text{S}}} \right) \, \times \, \left( {{\text{C1}}/{\text{t}}} \right),$$

where P is the apparent permeability, Vr is the volume of medium in the basolateral side of the chamber (Vr = 0.7 cm^3^), C0 is the concentration of the fluorescent dextran in the apical side of the transwell at t0 (C0 = 10 μg/ml), S is the surface area of the monolayer (S = 0.33 cm^2^), C1 is the concentration of the fluorescent dextran in the basolateral side of the chamber after incubation and t is the incubation time (t = 3600 s).

### ZO-1 immunofluorescence and imaging

After measuring endothelial cell permeability, bEnd3 cells on transwell inserts were washed three times in calcium- and magnesium-free phosphate buffered saline (PBS). Cells were then fixed in cold methanol/acetone (50%/50%) for 20 min at − 20 °C. After three rinses in PBS, cells were incubated with the blocking solution consisting of PBS supplemented with 5% FBS and 0.3% Triton X-100. Inserts were incubated with primary antibodies against zonula occludens-1 (ZO-1, dilution 1:100, Invitrogen Cat. #61-7300) overnight at 4 °C in a humidified chamber. Following three rinses in PBS, cells were incubated for 1 h in the dark with goat anti-rabbit Cy3 (dilution 1:500, Jackson ImmunoResearch) in combination with DAPI (dilution 1:1000, ThermoFisher Scientific). All antibodies were diluted in PBS supplemented with 2% BSA and 0.1% Triton X-100. Insert membranes were cut out with a scalpel, mounted on a glass slide with Prolong Gold Antifade and coated with a glass coverslip. Images were acquired using a 20X objective on an Axioskop2 plus microscope (Carl Zeiss) equipped with an Infinity 3S camera (Lumenera) and Infinity Analyze imaging software (Lumenera). The percentage of cells with altered tight junctions was measured by counting the number of cells with discontinued ZO-1 labelling around the cell, as a proportion of the total number of cells. Total number of cells analyzed = 833 and 597 for control IgG and anti- β1 integrin antibody, respectively. All in vitro experiments were performed twice, with similar results. For each experiment, all conditions were conducted in duplicate, and each permeability measurement was performed in triplicate. Data are represented as mean ± SEM. Statistical significance was assessed by unpaired, two-tailed Student’s t-test, in which p values < 0.05 were defined as statistically significant.

## Results

### Aged mice show greater hypoxic upregulation of cerebrovascular fibronectin and α5 integrin

**Fig. 1:**
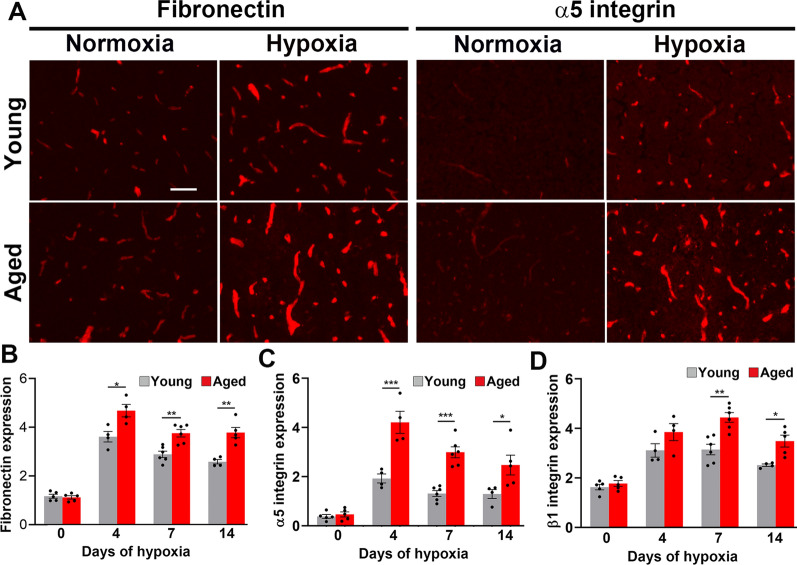
Chronic mild hypoxia (CMH)-induced upregulation of fibronectin and α5 integrin is greater in aged brain.** A** Frozen brain sections taken from young (8–10 weeks) or aged (20 months) mice exposed to normoxia or 7 days hypoxia (8% O_2_) were stained for fibronectin or α5 integrin. Images were captured in the midbrain. Scale bar = 50 μm. **B–D** Quantification of fibronectin (**B**), α5 integrin (**C**), or β1 integrin (**D**) in the midbrain after 0, 4, 7 and 14-days hypoxia. All results are expressed as the mean ± SEM (n = 4–6 mice/group). *p < 0.05, **p < 0.01, ***p < 0.001. Note that CMH-induced upregulation of fibronectin and α5 and β1 integrin was greater in aged brain

In a recent study we compared the hypoxia-induced cerebrovascular remodelling response in young (8–10 weeks) and aged (20 months) mice. Compared with young mice, aged mice showed delayed vascular maturation that was associated with greatly increased BBB breakdown [31]. As we have shown that the fibronectin-α5β1 integrin signalling axis promotes angiogenic remodelling in the CNS [18], we wondered if reduced activation of this pathway might account for the delayed vascular remodelling in aged mice. To answer this question, we compared cerebrovascular expression of fibronectin and α5 integrin in frozen brain sections derived from young (8–10 weeks) or aged (20 months) mice that had been exposed to normoxia or chronic mild hypoxia (CMH, 8% O_2_) for periods up to 14 days. As shown in Fig. 1A–C, hypoxia promoted marked upregulation of cerebrovascular fibronectin and α5 integrin in both young and aged mice, but surprisingly, the increases were noticeably greater in aged mice. Hypoxic upregulation of the β1 integrin subunit, the binding partner of α5, was also greater in aged mice (Fig. 1D). Figure 1 illustrates these findings in the midbrain and similar responses were observed in all brain regions examined. By comparison, expression levels of the vascular basement membrane protein laminin and its receptor α6β1 integrin were also upregulated by hypoxia but were not appreciably different between young and aged brains (Additional file 1). Vascular expression of the collagen receptor α1β1 integrin was not noticeably affected by hypoxia or age.

### β1 integrin blockade greatly increases hypoxia-induced BBB leak in both young and aged mice, but does not prevent hypoxia-induced endothelial proliferation or increased vascularity

**Fig. 2:**
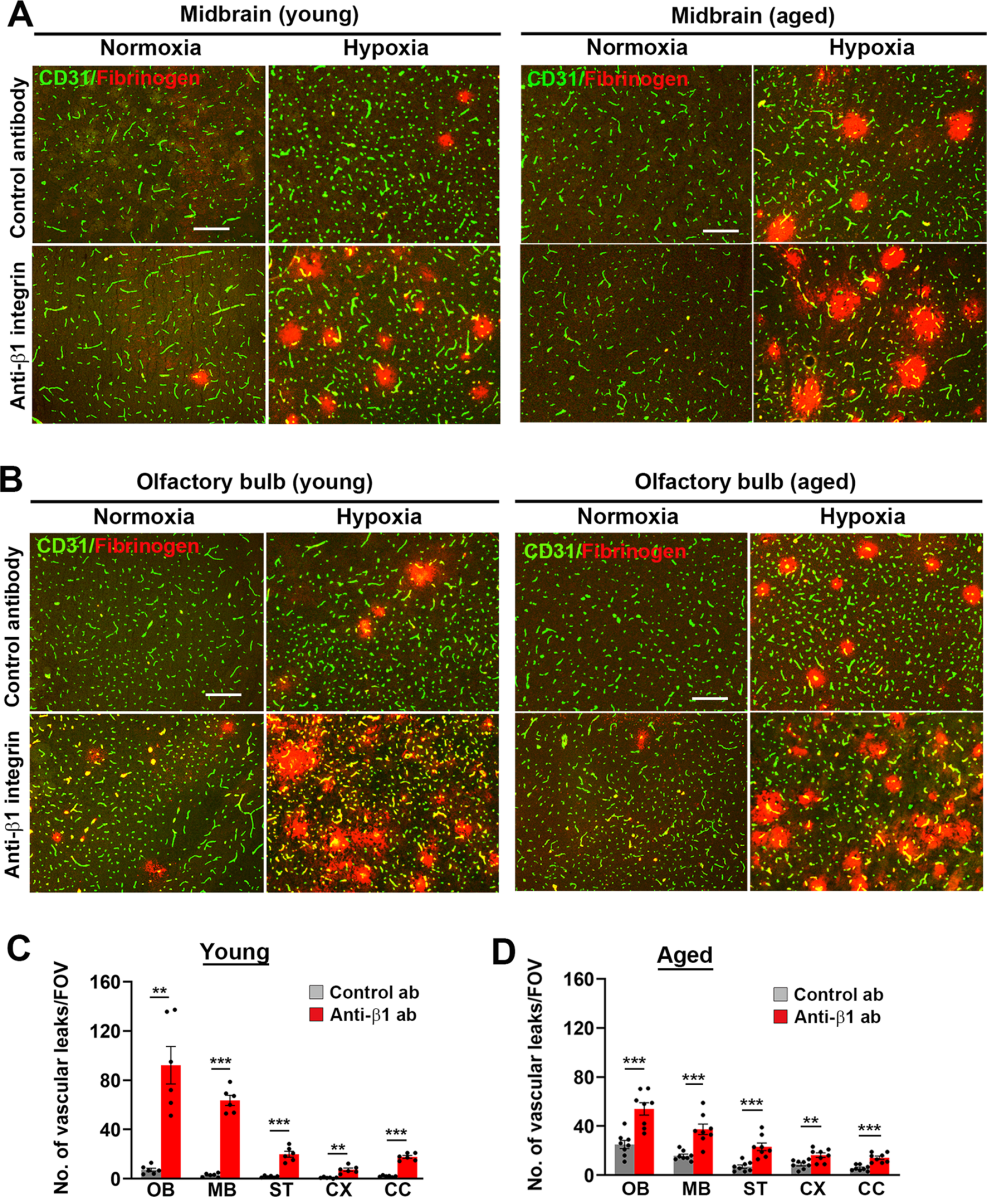
β1 integrin blockade greatly increases hypoxia-induced BBB breakdown in young and aged mice. Frozen brain sections taken from young (8–10 weeks) or aged (20 months) mice exposed to normoxia or hypoxia (8% O_2_) that had received daily intraperitoneal injections of the anti-mouse β1 integrin function-blocking antibody or isotype control antibody for 4 days were stained for CD31 (AlexaFluor-488) and fibrinogen (Cy-3). Images show the midbrain (**A**) or olfactory bulb (**B**). Scale bar = 200 μm. **C–D** Quantification of the number of vascular leaks/FOV in young (**C**) or aged (**D**) brain after 0- or 4-days hypoxia. Results are expressed as the mean ± SEM (n = 6–8 mice/group). **p < 0.01, ***p < 0.001. Note that β1 integrin blockade markedly increased the extent of hypoxia-induced vascular leak in all regions examined of both young and aged brain. OB, olfactory bulb; MB, midbrain; ST, striatum; CX, cerebral cortex; CC, corpus callosum

As our previous results demonstrate that compared to young brain, cerebrovascular endothelium in the aged brain is more activated by hypoxia, as shown by increased VCAM-1 and MECA-32 expression [31], this raises the possibility that increased expression of the fibronectin-α5β1 integrin signalling axis in aged brain could be driving increased endothelial activation and subsequent BBB disruption. This idea would be consistent with previous work by Hakanpaa et al. who showed that a blocking β1 integrin antibody reduced intestinal vascular leak in a mouse model of sepsis [25]. If this is also true of blood vessels in the CNS, then blocking α5β1 integrin function would stabilize the BBB and reduce vascular leak. To test this possibility, we evaluated BBB disruption in young (8–10 weeks) and aged (20 months) mice exposed to CMH for 4 days, that received daily intraperitoneal (i.p.) injections of either the anti-mouse β1 integrin function-blocking antibody HMβ1-1 or an isotype control antibody (at doses of 2.5 mg/kg). To validate that the blocking antibody reached the target site, we first performed immunofluorescence (IF) with an anti-hamster secondary antibody, and this confirmed that the β1 integrin blocking antibody strongly localized specifically to cerebral blood vessels (Additional file 1: Fig. S2). The increased fluorescent signal intensity observed under hypoxic versus normoxic conditions is consistent with the hypoxic upregulation of vascular β1 integrin expression shown in Fig. 1D. Vascular leak was evaluated by dual-IF using CD31 to label endothelial cells and fibrinogen to detect extravascular leak. As shown in Fig. 2A, B, under normoxic conditions, no vascular leak was detected in young or aged mice receiving the isotype control antibody. Interestingly, the impact of β1 integrin blockade under normoxic conditions differed between young and aged brains. In the aged brain, a small, almost negligible number of leaks were seen, but in the young brain, a significantly greater number of vascular leaks was triggered in almost all brain areas examined (Additional file 1: Fig. S3).

Because we recently showed that hypoxia-induced cerebrovascular leak in aged brain is much (five–tenfold) greater than in young brain [31], we expected that β1 integrin blockade would trigger much greater vascular breakdown in aged brain. However, as shown in Fig. 2A–D, much to our surprise, we found that the β1 integrin antibody greatly increased the number of hypoxia-induced vascular leaks both in the young and aged brain in all areas examined. Indeed, in the two brain areas most affected, the olfactory bulb and midbrain, the number of leaks triggered by the β1 integrin antibody in the young brain was significantly higher than the aged (90.8 ± 11.8 vs. 54.4 ± 5.2 leaks/FOV, p < 0.05, and 65.3 ± 5.4 vs. 35.4 ± 3.5 leaks/FOV, p < 0.01, respectively; see Fig. 2C, D).

**Fig. 3:**
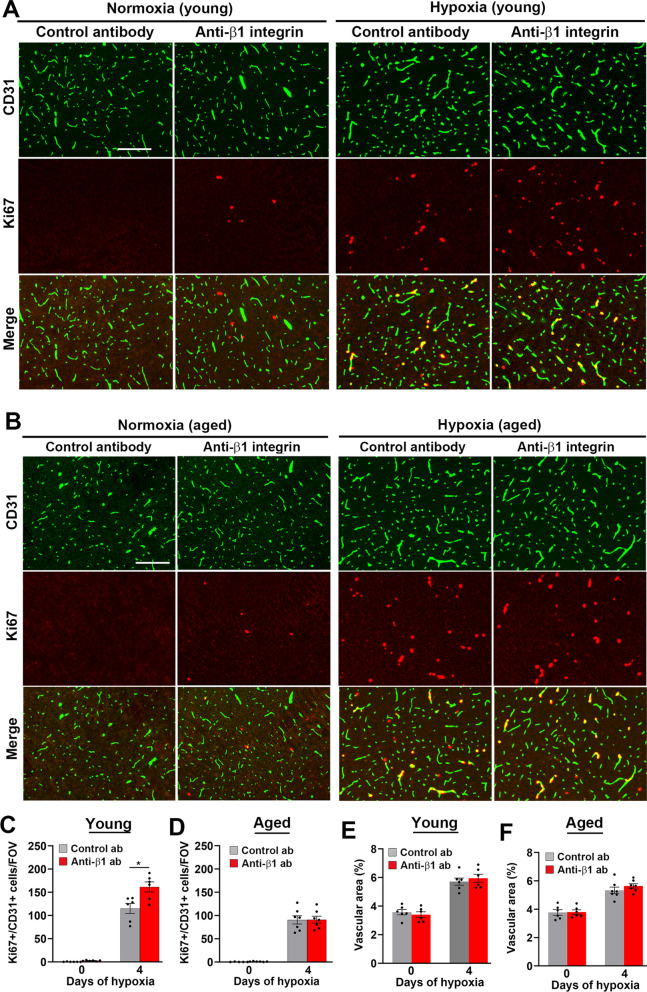
β1 integrin blockade does not prevent hypoxia-induced endothelial proliferation or increased vascularity in young or aged brain. Frozen brain sections taken from young (8–10 weeks) and aged (20 months) mice exposed to normoxia or hypoxia (8% O_2_) that had received daily intraperitoneal injections of the anti-mouse β1 integrin function-blocking antibody or isotype control antibody for 4 days were stained for CD31 (AlexaFluor-488) and the proliferation marker Ki67 (Cy-3). Images show the midbrain in young (**A**) or aged (**B**) mice. Scale bar = 200 μm. **C–F**. Quantification of the number of proliferating endothelial cells (CD31 + /Ki67 + cells)/FOV (**C–D**), or vascular area (% of total) (**E–F**) after 0- or 4-days hypoxia. Results are expressed as the mean ± SEM (n = 6–7 mice/group). *p < 0.05. Note that β1 integrin blockade greatly did not prevent hypoxia-induced endothelial proliferation or increased vascularity, and unexpectedly, increased hypoxia-induced endothelial proliferation in young brain. *OB* olfactory bulb, *MB* midbrain, *ST* striatum, *CX* cerebral cortex, *CC* corpus callosum

As previous data suggest an instructive role for α5β1 integrin in driving hypoxia-induced endothelial proliferation and angiogenesis in the CNS [18], we next examined how the anti-β1 integrin antibody impacts endothelial proliferation in young and aged brain by performing dual-IF with CD31 and the proliferation marker Ki67. Consistent with previous findings, endothelial proliferation at both ages was negligible under normoxic conditions but strongly increased with hypoxia (Fig. 3A–D) [33]. However, contrary to our expectation that β1 integrin blockade would inhibit endothelial proliferation, the rate of endothelial proliferation in aged brain was not affected by the anti-β1 integrin antibody and in young brain, it was surprisingly marginally increased. In keeping with these findings, β1 integrin blockade had no noticeable effect on preventing the increased vascularity stimulated by CMH either in young or aged brain (Fig. 3E, F, respectively). In summary, these findings demonstrate that both in young and aged brain, while β1 integrin blockade had little impact on the angiogenic response triggered by CMH, it profoundly reduced the stability of the newly formed blood vessels, resulting in much greater levels of BBB disruption at both ages.

Because we know from our previous studies that the extent of hypoxia-induced vascular leak peaks between 4 and 7 days CMH [29], we also looked at an earlier timepoint to examine if 2 days CMH was sufficient to trigger vascular leak in aged brain and whether β1 integrin blockade impacts vascular disruption at this earlier timepoint. As shown in Additional file 1: Fig. S4, this revealed several important points. First, after 2 days CMH, the extent of vascular leak in the olfactory bulb was much greater than any other brain region including the midbrain (which showed very few at this early timepoint; Additional file 1: Fig. S4A, C), demonstrating that the olfactory bulb is by far the most vulnerable brain area for hypoxia-induced vascular leak. Second, even at the early 2-day timepoint, β1 integrin blockade significantly increased the number of vascular leaks in all brain regions examined. Third, while an obvious number of vascular leaks were present in the olfactory bulb at this early timepoint, proliferating endothelial cells were very scarce (Additional file 1: Fig. S4B, D), demonstrating that vascular breakdown occurs well in advance of endothelial proliferation.

**Fig. 4:**
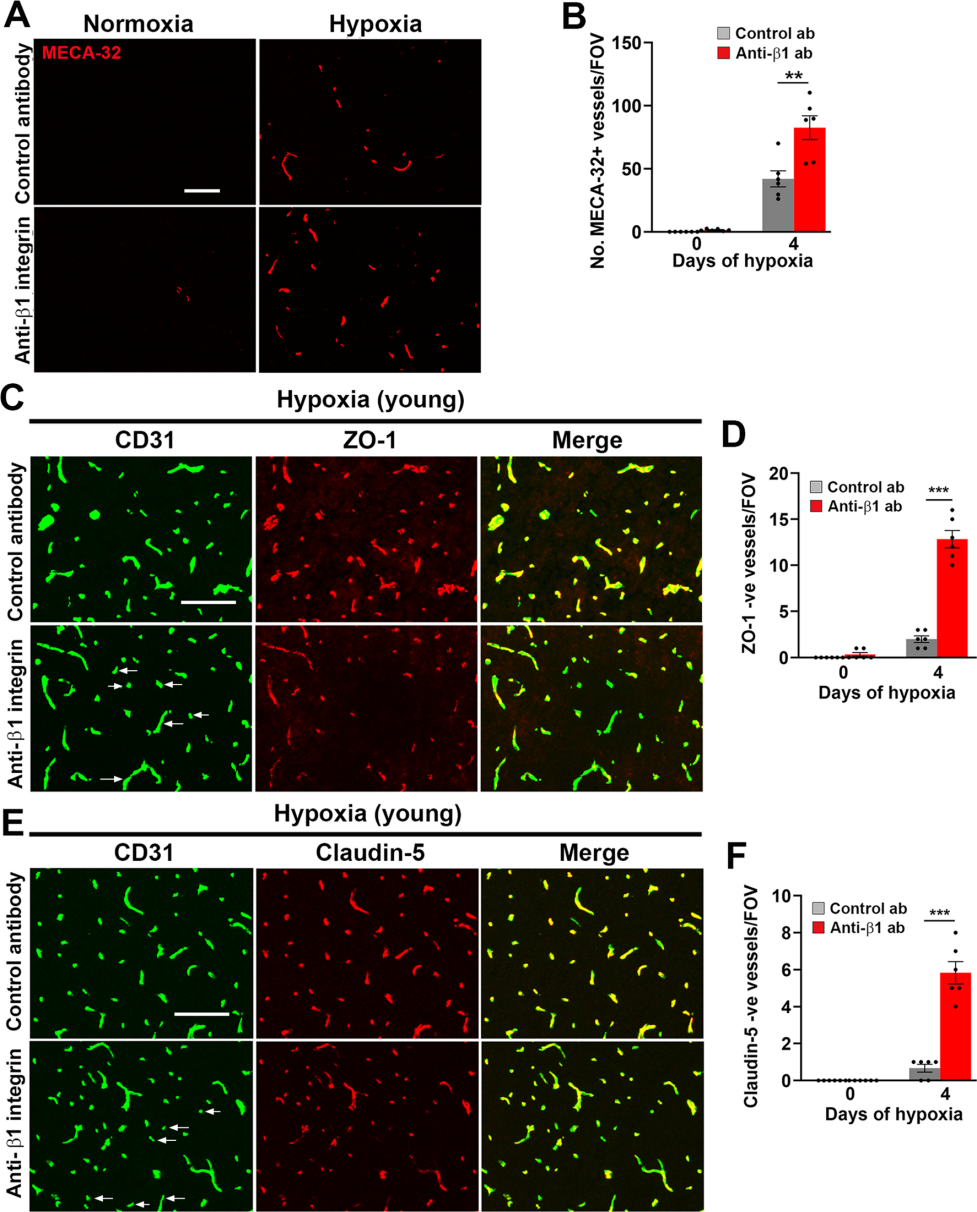
β1 integrin blockade increases cerebrovascular expression of MECA-32 and enhances loss of tight junction proteins in young mice under hypoxic conditions. **A**, **C, E** Frozen brain sections taken from young (8–10 weeks) mice exposed to normoxia or hypoxia (8% O_2_) that received daily intraperitoneal injections of the anti-mouse β1 integrin function-blocking antibody or isotype control antibody for 4 days were stained for MECA-32 (**A**), CD31 (AlexaFluor-488) and ZO-1 (Cy-3; **C**), or CD31 (AlexaFluor-488) and claudin-5 (Cy-3; **E**). All images were captured in the midbrain. Scale bar = 100 µm. **B, D, F** Quantification of the number of MECA-32 + vessels/FOV (**B**), or number of vessels/FOV lacking ZO-1 (**D**) or claudin-5 (**F**). Results are expressed as the mean ± SEM (n = 6 mice/group). **p < 0.01, ***p < 0.001. Note that β1 integrin blockade increased cerebrovascular expression of MECA-32 and greatly enhanced loss of endothelial tight junction proteins in young mice under hypoxic conditions

Angiogenesis can be broken down into distinct stages that includes separation of neighboring endothelial cells, endothelial proliferation, migration, differentiation, and finally maturation of new vessels. As the β1 integrin antibody appears not to inhibit endothelial proliferation or migration (as it had no impact on hypoxia-induced endothelial proliferation or increased vascularity), this suggests that it enhances vascular leak by interfering with the final stages of vessel maturation. To directly examine this, we evaluated in young brain the impact of the β1 integrin antibody on endothelial expression of MECA-32, a marker of immature/remodelling cerebral blood vessels [34, 35]. Consistent with previous observations, no MECA-32 expression was detected under normoxic conditions, but under hypoxic conditions, the β1 integrin antibody greatly increased the number of MECA-32-positive blood vessels compared to mice receiving isotype control antibody (Fig. 4A, B). This supports the concept that β1 integrin inhibition results in greater hypoxia-induced vascular breakdown because it delays the maturation of newly formed blood vessels. As vascular leak is associated with loss of endothelial tight junction proteins [5, 32], we next examined how β1 integrin blockade influences the loss of ZO-1 and claudin-5. As expected, under normoxic conditions, all cerebral blood vessels expressed high levels of ZO-1 and claudin-5. While hypoxia triggered some small degree of loss of tight junction proteins in mice treated with control antibody, this loss was greatly enhanced in mice treated with the β1 integrin antibody (Fig. 4C–F (dual-IF) and Additional file 1: Fig. S5 (triple-IF); arrows denote loss of tight junction protein). As previous work has shown that β1 integrin controls the localization of VE-cadherin at endothelial cell–cell junctions [28], we also examined if β1 integrin blockade perturbs this localization by performing triple-IF with CD31/fibrinogen/VE-cadherin. This showed that while most blood vessels expressed strong levels of VE-cadherin, vessels at the center of vascular leaks showed greatly reduced levels of VE-cadherin (Additional file 1: Fig. S6).

### β1 integrin blockade switches the hypoxic response of microglia in young brain towards that of the aged

**Fig. 5:**
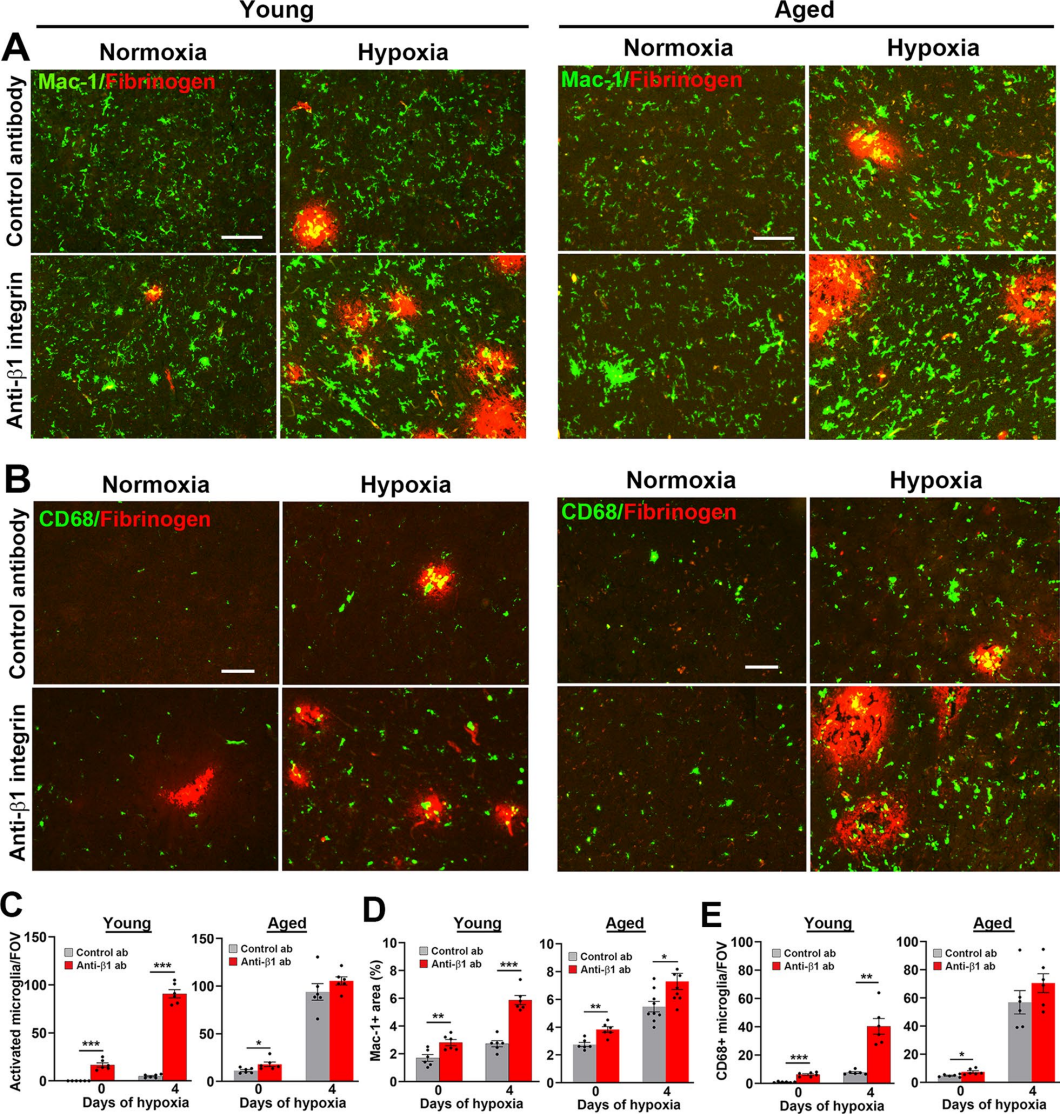
β1 integrin blockade greatly enhances microglial activation in the hypoxic young but not aged brain. Frozen brain sections taken from young (8–10 weeks) and aged (20 months) mice exposed to normoxia or hypoxia (8% O_2_) that received daily intraperitoneal injections of the anti-mouse β1 integrin function-blocking antibody or isotype control antibody for 4 days were stained for Mac-1 (AlexaFluor-488) and fibrinogen (Cy-3) (**A**) or CD68 (AlexaFluor-488) and fibrinogen (Cy-3) (**B**). Images were captured in the midbrain. Scale bars = 100 μm. Quantification of the number of morphologically activated microglia/FOV (**C**), total Mac-1 area/FOV (**D**) or number of CD68 + microglia/FOV (**E**) after 0- or 4-days hypoxia. Results are expressed as the mean ± SEM (n = 6–9 mice/group). *p < 0.05, **p < 0.01, ***p < 0.001. Note that β1 integrin blockade strongly increased all parameters of microglial activation in the young hypoxic brain, but much less so in the aged brain

**Fig. 6:**
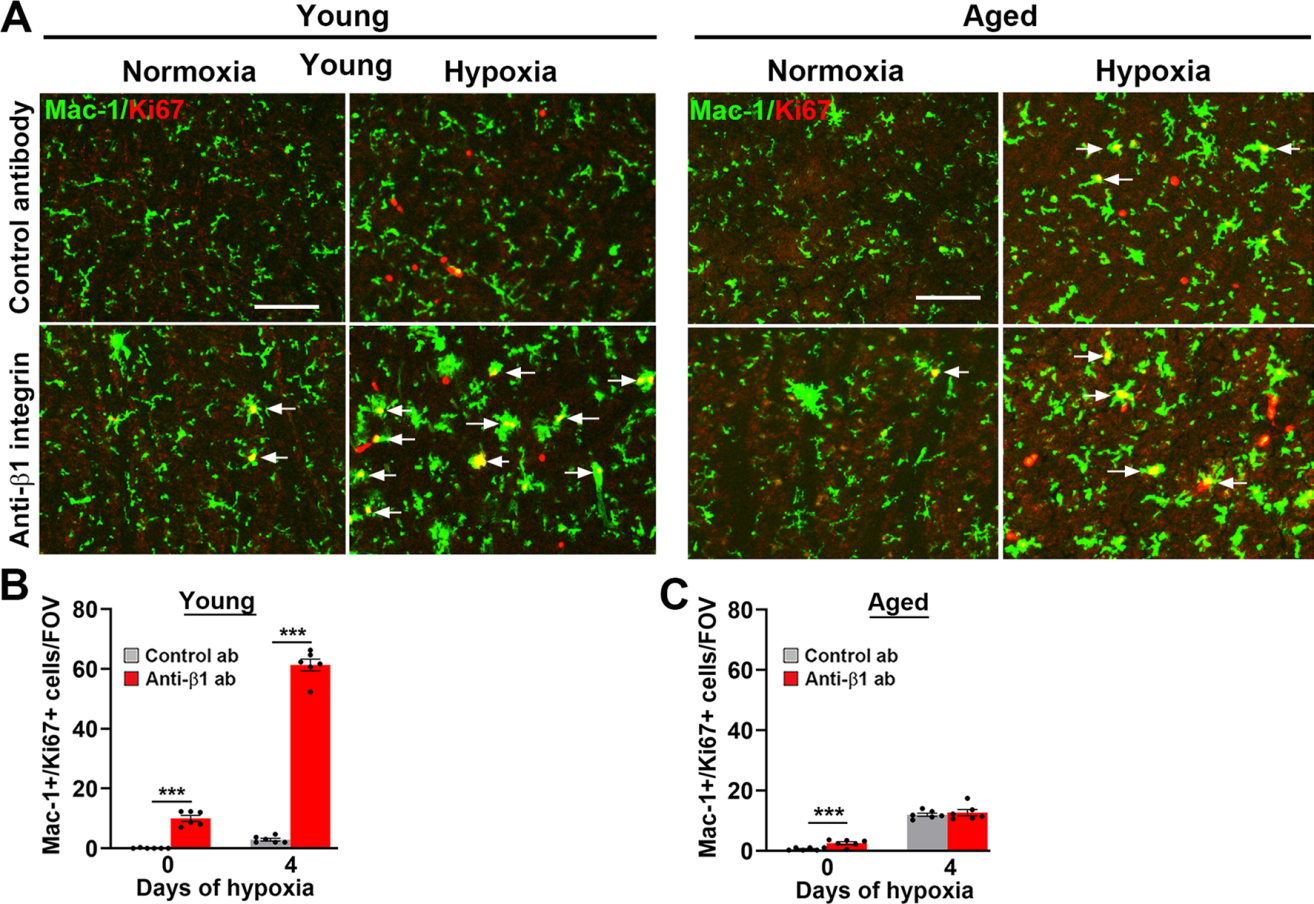
β1 integrin blockade strongly stimulated microglial proliferation in the hypoxic young but not aged brain. Frozen brain sections taken from young (8–10 weeks) and aged (20 months) mice exposed to normoxia or hypoxia (8% O_2_) that received daily intraperitoneal injections of the anti-mouse β1 integrin function-blocking antibody or isotype control antibody for 4 days were stained for Mac-1 (AlexaFluor-488) and Ki67 (Cy-3) (**A**). Images were captured in the midbrain. Scale bar = 100 μm. Quantification of the number of Mac-1+/Ki67+ cells/FOV in young (**B**) or aged (**C**) brain after 0- or 4-days hypoxia. Results are expressed as the mean ± SEM (n = 6 mice/group). ***p < 0.01. Note that β1 integrin blockade strongly increased microglial proliferation in the young hypoxic brain, but not in the aged brain

Recently, we described some fundamental differences in microglial behaviour between young and aged brain [31]. In young brain, microglia occupy a low state of activation under normoxic conditions and are not noticeably activated by hypoxia, except for those few microglia that are close to leaky blood vessels. In contrast, microglia in aged brain occupy a higher state of activation, even under normoxic conditions, and all microglia throughout the brain show a strong activation response to hypoxia. To compare the impact of β1 integrin blockade on microglial activation at different ages, we performed dual-IF on brain sections from young and aged mice for Mac-1/fibrinogen, CD68/fibrinogen, and Mac-1/Ki67. This revealed that under normoxic conditions, in both young and aged mice, β1 integrin blockade triggered relatively small but significant increases in microglial activation as shown by an increased number of morphologically activated cells (larger cell body and thick short process extensions; Fig. 5A). Under hypoxic conditions, β1 integrin blockade had a much stronger effect in young mice, triggering widespread rampant microglial activation as shown by (i) higher expression of Mac-1 and a morphological switch into the activated phenotype (Fig. 5A, C, D), (ii) increased number of microglia expressing the activation marker CD68 (Fig. 5B, E), and (iii) a striking increase in microglial proliferation (Mac-1/Ki67 dual-positive cells; Fig. 6A, see arrows and Fig. 6B). By contrast, the impact of β1 integrin blockade on microglial activation in the aged hypoxic brain was much less compared to the young hypoxic brain (Figs. 5 and 6). This is best illustrated by the observation that the number of proliferating microglia triggered by β1 integrin blockade was strongly increased in young brain (Fig. 6A, B) but was not affected at all in aged brain (Fig. 6A, C). We postulate that the most likely reason for this is that microglia in the aged hypoxic brain are probably already close to the maximum level of activation, so β1 integrin blockade has little impact on enhancing their activation, while microglia in the young hypoxic brain are nowhere near their ceiling of activation. In summary, these findings demonstrate that β1 integrin blockade of young mice under hypoxic conditions, triggers greatly increased vascular leak that is accompanied by an equally robust microglial activation response. In other words, β1 integrin blockade switches the BBB phenotype of the young brain towards that of the aged.

### β1 integrin blockade decreased the integrity of a brain endothelial monolayer

**Fig. 7:**
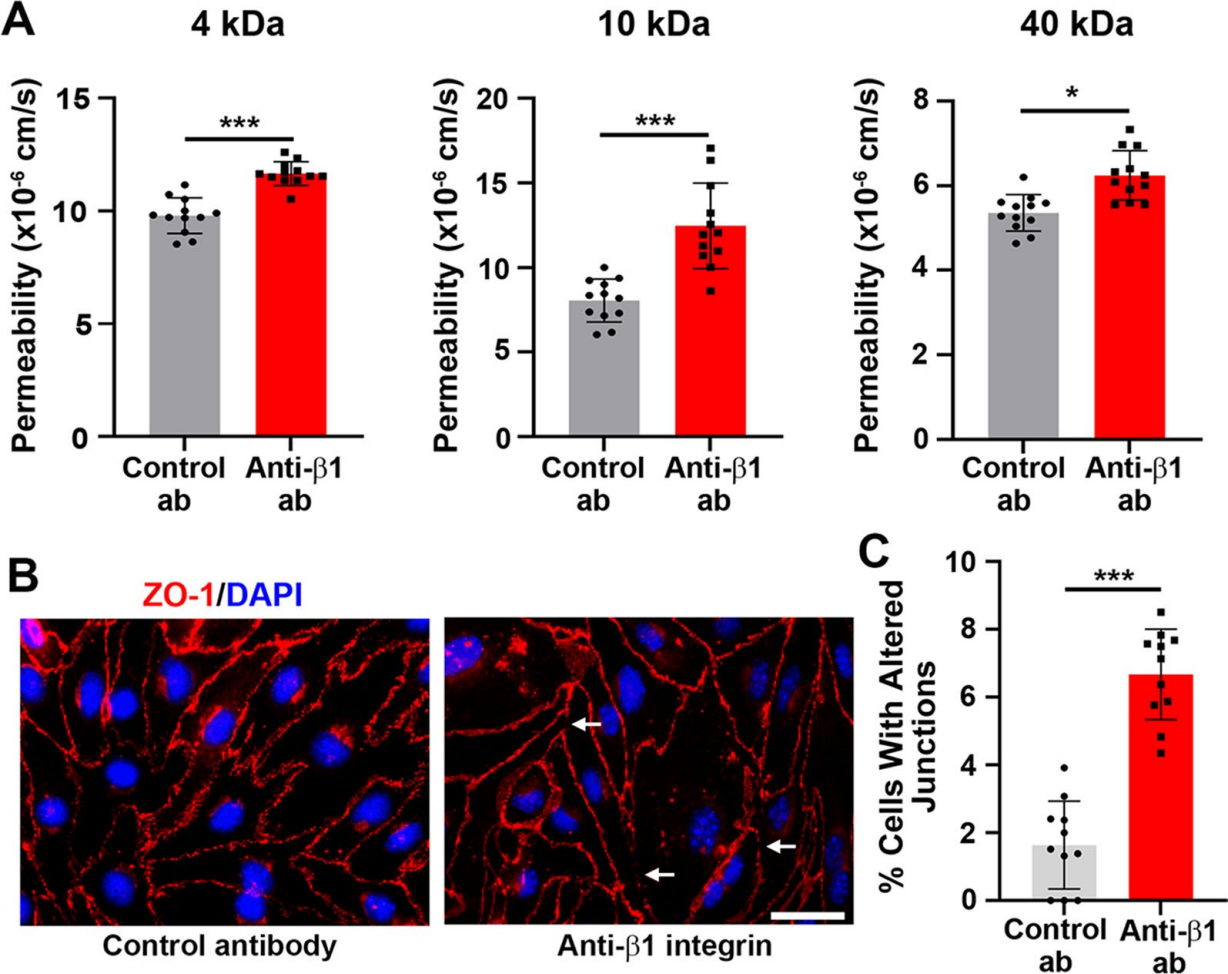
β1 integrin blockade reduced the integrity of an endothelial monolayer. **A** A brain endothelial cell monolayer of bEnd3 cells in transwell cultures was measured 48 h after incubation with 10 µg/ml control antibody or anti-β1 integrin antibody. Permeability was measured with 4 kDa FITC-conjugated Dextran, 10 kDa Cascade Blue-conjugated Dextran and 40 kDa Texas Red-conjugated Dextran. *p < 0.05 and ***p < 0.001. **B** Representative images of ZO-1 immunostaining (red) and DAPI nuclear staining (blue) in endothelial cells after being incubated with control or anti-β1 integrin antibody. Scale bar = 25 μm. White arrows indicate points of junction disruption. **C** Quantification of the percentage of cells with disrupted junctions. ***p < 0.001. Note that β1 integrin blockade significantly increased the permeability of the endothelial monolayer to all three sizes of dextrans and induced disruptions in the ZO-1 staining pattern

To further examine the role of β1 integrins in maintaining vascular integrity, we also examined this in vitro. Endothelial cell monolayers of the brain endothelial cell line bEnd3 were established, then control or anti-β1 antibodies were introduced into the culture medium. Two days later, the permeability of the endothelial cell monolayer was measured using three different sizes of fluorescently labelled dextrans (4, 10 and 40 kDa). This revealed that β1 integrin blockade significantly increased the permeability of the endothelial monolayer to all three sizes of dextrans (Fig. 7A). Furthermore, when we performed immunostaining of the tight junction protein ZO-1, we noticed that β1 integrin blockade induced disruptions in the ZO-1 staining pattern, and quantification showed that cells treated with the β1 integrin antibody had significantly more disruptions than cells treated with control antibody (Fig. 7B, C).

## Discussion

In this study we examined the impact of blocking β1 integrin function on blood–brain barrier (BBB) integrity, both under normoxic conditions, when the BBB is stable, and during hypoxic conditions when an extensive vascular remodeling response occurs. Based on our findings, several conclusions can be drawn. First, β1 integrins play an essential role in maintaining BBB integrity, both under stable normoxic conditions and during hypoxia-induced vascular remodeling. Second, while the BBB-disruptive impact of β1 integrin blockade is strongest during active vascular remodeling, β1 integrin function is also required to maintain BBB integrity during stable (normoxic) conditions. Third, the impact of β1 integrin blockade was more pronounced in younger mice, consistent with our observation that the rate of vascular remodeling is greater in younger mice. Fourth, as β1 integrin blockade in young mice promotes large, parallel increases in vascular leak and microglial activation, this supports the notion that decreased BBB integrity leads directly to enhanced microglial activation. In this regard, it appears that β1 integrin blockade switches the cerebrovascular phenotype of young mice towards that of aged mice, i.e., greater vascular leak leads to enhanced microglial activation. These findings suggest that enhancing β1 integrin function at the aged BBB may hold therapeutic potential by reverting the deteriorating BBB phenotype back towards that of the young.

### β1 integrins support BBB stability

The most important finding from our studies is that while β1 integrins appear to be dispensable for most stages of vascular remodeling, including endothelial proliferation and migration, as indicated by lack of impact of blockade on hypoxia-induced increased vascularity, they play an essential role in promoting endothelial organization into mature blood vessels with high integrity. These findings are consistent with previous work showing that genetic deletion of endothelial β1 integrin leads to loss of VE-cadherin localization at endothelial-endothelial junctions, resulting in leaky blood vessels [28]. Consistent with this, Osada et al. showed that inhibiting β1 integrin function increased vascular permeability of brain endothelium both in vitro and in vivo [27], and later confirmed this by demonstrating that transgenic mice with reduced endothelial β1 integrins also showed increased BBB disruption [26]. Interestingly, other studies found conflicting results. In a mouse model of endotoxemia (sepsis), Hakanpaa et al. showed that β1 integrin blockade (using the same antibody and dose used in the current study) suppressed vascular leak in the lungs, suggesting that β1 integrin blockade stabilized vascular integrity in the lungs [25]. Furthermore, the Bix lab demonstrated that transgenic mice lacking α5β1 integrin or mice treated with a peptide inhibiting α5β1 integrin (ATN-161) were largely protected against BBB breakdown in a mouse model of ischemic stroke [9, 24]. What could account for these fundamentally different observations? Notably, studies performed under non-pathological conditions consistently show that β1 integrin inhibition (either by blocking antibody or genetic deletion) result in reduced vascular integrity, implying that β1 integrins are important mediators of vascular integrity, as one would expect from their critical role in cell adhesion [36, 37]. By contrast, studies performed under pathological conditions (sepsis or ischemic stroke) appear to show that β1 integrin inhibition protected vascular integrity. We postulate that the most likely reason for this is that under disease conditions, the primary impact of the blocking β1 integrin antibody will be to block the function of inflammatory leukocytes, thereby reduced leukocyte adhesion, extravasation, and migration within target tissue, resulting in less inflammation, which if true, would have the net effect of protecting vascular stability.

Given previous data supporting important roles for β1 integrins in driving endothelial proliferation and migration [18, 38, 39], it was somewhat surprising that β1 integrin blockade in this study did not attenuate these steps of the angiogenic sequence. However, studies in knockout mice have shown that β1 integrins are not always essential for endothelial proliferation and migratory events at specific parts of the vascular tree or at certain times [28]. In other words, the requirement for β1 integrins appears to be context dependent. Interestingly, while β1 integrin blockade had no impact on endothelial proliferation in aged brain, in the young brain it surprisingly increased proliferation. Of note, a similar effect was reported in endothelial β1 integrin-deficient mice [28]. Why this happens is unknown, though we suggest three possibilities: (i) by preventing endothelial terminal differentiation, β1 integrin blockade increases the available pool of mitogenic endothelial cells, (ii) the system senses the increased vascular leak and drives harder to plug these leaks by producing more endothelial cells, and (iii) the rampant microglial activation response triggered by vascular leak [29] produces angiogenic factors which stimulate endothelial proliferation [40].

Motivated by our observation that hypoxic-induction of the fibronectin-α5β1 integrin signaling axis was more strongly upregulated in the aged brain, it seemed plausible that blocking this mechanism might lead to a more stable BBB. However, we found the exact opposite. We speculate that in the face of a delayed vascular remodeling response in aged brain, increased activation of the fibronectin-α5β1 integrin signaling pathway represents a compensatory mechanism designed to speed up angiogenesis. Previous studies have shown that within the brain, β1 integrins are expressed at high levels by endothelial cells, commensurate with their close proximity to the ECM-rich vascular basement membrane but are practically undetectable on other cell types [19, 41, 42]. Our current findings are consistent with this because the β1 integrin function-blocking antibody localized strongly to blood vessels but was undetectable on parenchymal tissue. Based on these observations, we believe that the primary target of the function-blocking β1 integrin antibody was endothelial cells, and that inhibition of endothelial β1 integrin function directly triggered vascular breakdown, which led to the release of blood-derived factors into the parenchyma, causing activation of microglia. However, other cells of the BBB, such as pericytes and astrocytes, also express β1 integrins [43–46] albeit at much lower levels than endothelial cells, and therefore we cannot rule out the possibility that inhibition of β1 integrin function on these cell types may have also contributed to the vascular disruption.

### The effects of β1 integrin blockade were more profound in young brain

Another striking finding was that β1 integrin blockade had a much greater impact on hypoxia-induced BBB disruption in young brain. This was surprising given that hypoxia-induced vascular leak is five–tenfold greater in the aged brain [31], consistent with the notion that the BBB deteriorates with age [10, 11]. Yet levels of BBB disruption triggered by β1 integrin blockade in the young hypoxic brain were generally higher in all brain regions compared to aged brain. This was also notable in the normoxic brain, where BBB disruption levels were much less than hypoxia, but still markedly higher in the young brain. These findings are consistent with the idea that blood vessels in the young brain are more physiologically dynamic [31], i.e., they are faster to mount an angiogenic response to hypoxia and even under normoxic stable conditions, low levels of vascular remodeling are still ongoing as endothelial cells are continually striving to maintain the highest levels of BBB integrity. This would provide more opportunity for the β1 integrin blocking antibody to interfere with newly forming cell–matrix adhesive interactions, and thus manifest as greater levels of vascular leak in the young brain.

### What does this tell us about the relationship between vascular integrity and microglial activation?

Compared to young brain, the aged brain displays five–tenfold greater number of hypoxia-induced vascular leaks, and this correlates closely with much higher levels of microglial activation [31]. This raises the fundamental question: does vascular leak lead to microglial activation or vice versa? Our findings suggest this is most likely a two-way street. First, our recent study demonstrates that microglial activation can influence vascular integrity because when microglial activation in aged brain is attenuated with minocycline, this reduces the extent of BBB disruption [31]. Second, the findings from our current study suggest that the former is also true, by demonstrating that pharmacologically induced BBB disruption, particularly in young brain, leads to greatly enhanced microglial activation. In this regard, it appears that β1 integrin blockade switches the cerebrovascular phenotype of young mice towards that of aged mice, i.e., greater vascular leak leads to enhanced microglial activation. These findings suggest that enhancing β1 integrin function at the aged BBB may hold therapeutic potential by reverting the deteriorating BBB phenotype back towards that of the young.

## Conclusions

In this study we demonstrate that β1 integrins play an essential role in maintaining BBB integrity, both under stable normoxic conditions and to an even greater extent during hypoxia-induced vascular remodeling. Surprisingly, β1 integrin blockade did not reduce hypoxia-induced endothelial proliferation, nor did it prevent the hypoxia-associated increase in vascularity. Notably, the impact of β1 integrin blockade on triggering vascular disruption and the associated microglial activation, was more pronounced in younger mice, consistent with our observation that the rate of vascular remodeling is greater in younger mice. In this regard, it appears that β1 integrin blockade switches the cerebrovascular phenotype of young mice towards that of aged mice, i.e., greater vascular leak leading to enhanced microglial activation. Taken together, our findings suggest that enhancing β1 integrin function at the aged BBB may hold therapeutic potential by reverting the deteriorating BBB phenotype back towards that of the young.

## Supplementary Information

**Additional file 1: Figure S1** Chronic mild hypoxia (CMH)-induced upregulation of laminin and α6 and α1 integrins is similar in young and aged brains. **Figure S2**. Vascular localization of the function-blocking β1 integrin antibody in young and aged mice brains under normoxic and hypoxic conditions. **Figure S3**. The impact of β1 integrin blockade on cerebrovascular leak in young and aged mice under normoxic conditions. **Figure S4**. The impact of β1 integrin blockade on cerebrovascular leak and remodeling in aged mice after 2 days hypoxia. **Figure S5**. The impact of β1 integrin blockade on vascular tight junction protein expression. **Figure S6.** The impact of β1 integrin blockade on vascular VE-cadherin expression.

## Data Availability

The datasets used and/or analysed during the current study are available from the corresponding author upon reasonable request.

## Abbreviations

BM: Basement membrane
BBB: Blood–brain barrier
CMH: Chronic mild hypoxia
ECM: Extracellular matrix
FOV: Field of view
IF: Immunofluorescence
MECA-32: Mouse endothelial cell antigen-32
VCAM-1: Vascular cell adhesion molecule-1
VCID: Vascular contributions to cognitive impairment and dementia
ZO-1: Zonula occludens-1
