# Supplementary material for: β1 integrin is essential for blood–brain barrier integrity under stable and vascular remodelling conditions; effects differ with age
Source: Fluids Barriers CNS. 2023 Jul 3;20:52. doi: 10.1186/s12987-023-00453-0 (PMC10316581; doi:10.1186/s12987-023-00453-0)
Supplement: Supplementary file 1 — Additional file 1: Figure S1 Chronic mild hypoxia (CMH)-induced upregulation of laminin and α6 and α1 integrins is similar in young and aged brains. Figure S2. Vascular localization of the function-blocking β1 integrin antibody in young and aged mice brains under normoxic and hypoxic conditions. Figure S3. The impact of β1 integrin blockade on cerebrovascular leak in young and aged mice under normoxic conditions. Figure S4. The impact of β1 integrin blockade on cerebrovascular leak and remodeling in aged mice after 2 days hypoxia. Figure S5. The impact of β1 integrin blockade on vascular tight junction protein expression. Figure S6. The impact of β1 integrin blockade on vascular VE-cadherin expression. [file 12987_2023_453_MOESM1_ESM.pdf]

Additional file 1:

β1 integrin is essential for blood-brain barrier integrity under stable and vascular remodelling conditions; effects differ with age

Sebok K. Halder, Violaine D. Delorme-Walker and Richard Milner

Email: rmilner@sdbri.org

This PDF file includes:

Figures S1 to S6

Figure S1

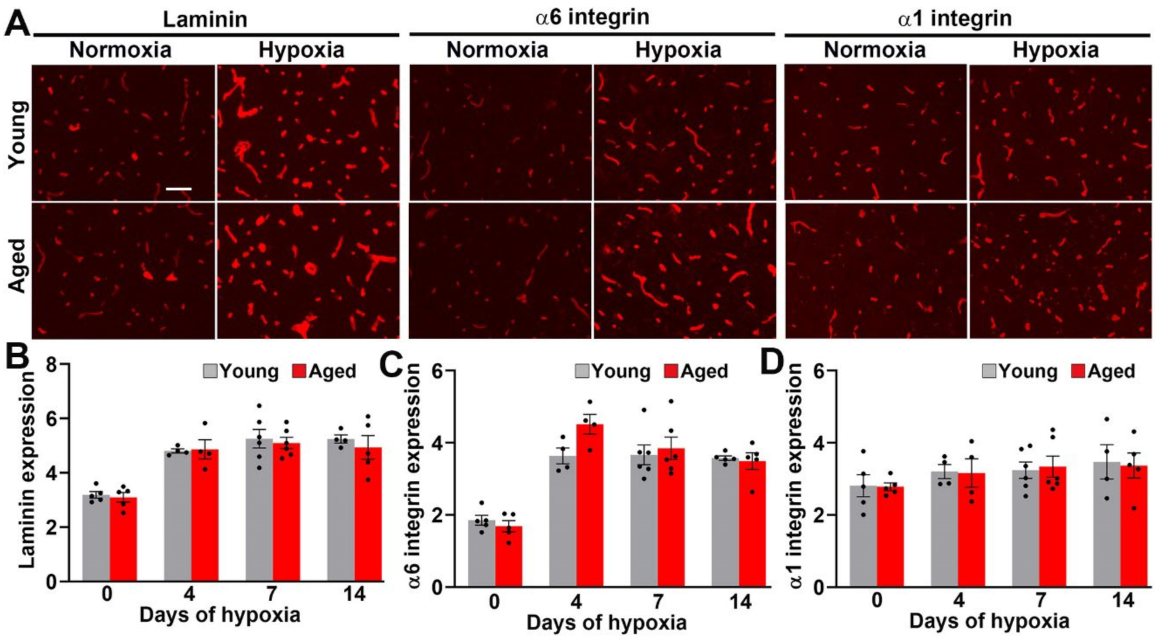

Figure S1. Chronic mild hypoxia (CMH)-induced upregulation of laminin and α6 and α1 integrins is similar in young and aged brains. A. Frozen brain sections taken from young (8-10 weeks) or aged (20 months) mice exposed to normoxia or 7 days hypoxia (8% O_2) were stained for laminin and the α6 and α1 integrins. Images were captured in the midbrain. Scale bar = 50 μm. **B-D.** Quantification of laminin **(B),** α6 integrin **(C),** or α1 integrin **(D)** in the midbrain after 0, 4, 7 and 14-days hypoxia. All results are expressed as the mean ± SEM (n = 4-6 mice/group). Note that CMH-induced upregulation of laminin and α6 and α1 integrins is similar in young and aged brains.

Figure S2

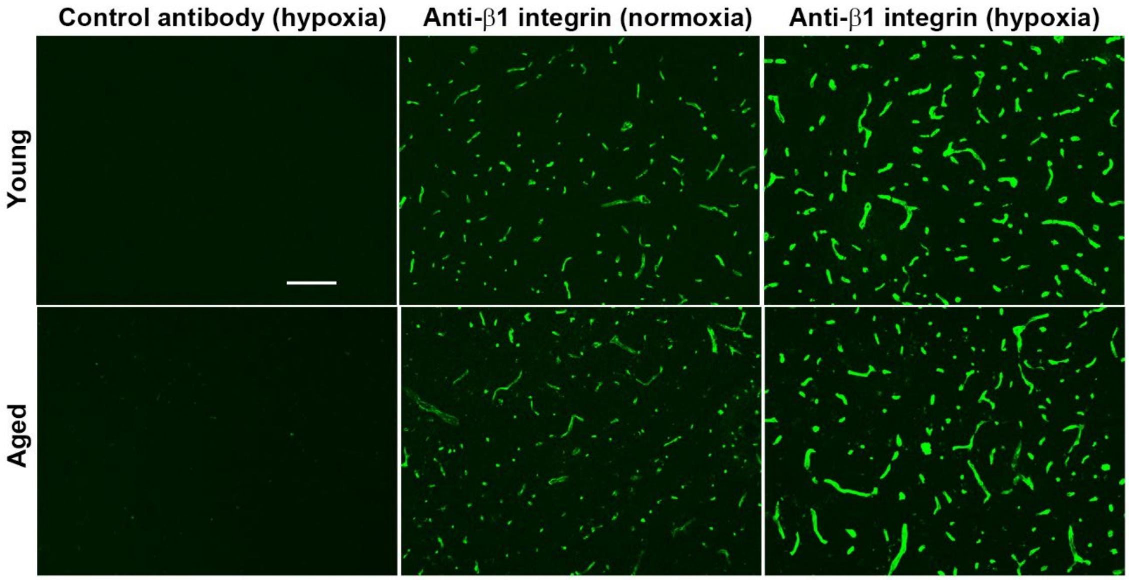

Figure S2. Vascular localization of the function-blocking β1 integrin antibody in young and aged mice brains under normoxic and hypoxic conditions. Frozen brain sections taken from young (8-10 weeks) and aged (20 months) mice exposed to normoxia or hypoxia (8% O_2) for 4 days that received daily intraperitoneal (i.p.) injections of either the anti-mouse β1 integrin function-blocking antibody HMβ1-1 or an isotype control antibody (at doses of 2.5 mg/kg) were stained with an anti-hamster secondary antibody. Scale bar = 100 μm. Note that the β1 integrin blocking antibody strongly localized to cerebral blood vessels and that fluorescent signal intensity increased under hypoxic conditions, consistent with the hypoxic upregulation of vascular β1 integrin expression as shown in Figure 1D.

Figure S3

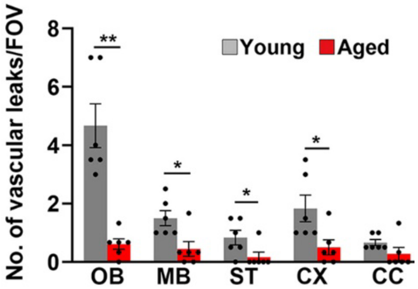

Figure S3. The impact of β1 integrin blockade on cerebrovascular leak in young and aged mice under normoxic conditions. Quantification of the number of vascular leaks/FOV following 4-days of β1 integrin blockade. Results are expressed as the mean ± SEM (n = 6 mice/group). * $p < 0.05$, ** $p < 0.01$. Note that under normoxic conditions, β1 integrin blockade triggered a greater number of vascular leaks in almost all regions of young brain compared to aged brain. OB, olfactory bulb; MB, midbrain; ST, striatum; CX, cerebral cortex; CC, corpus callosum.

Figure S4

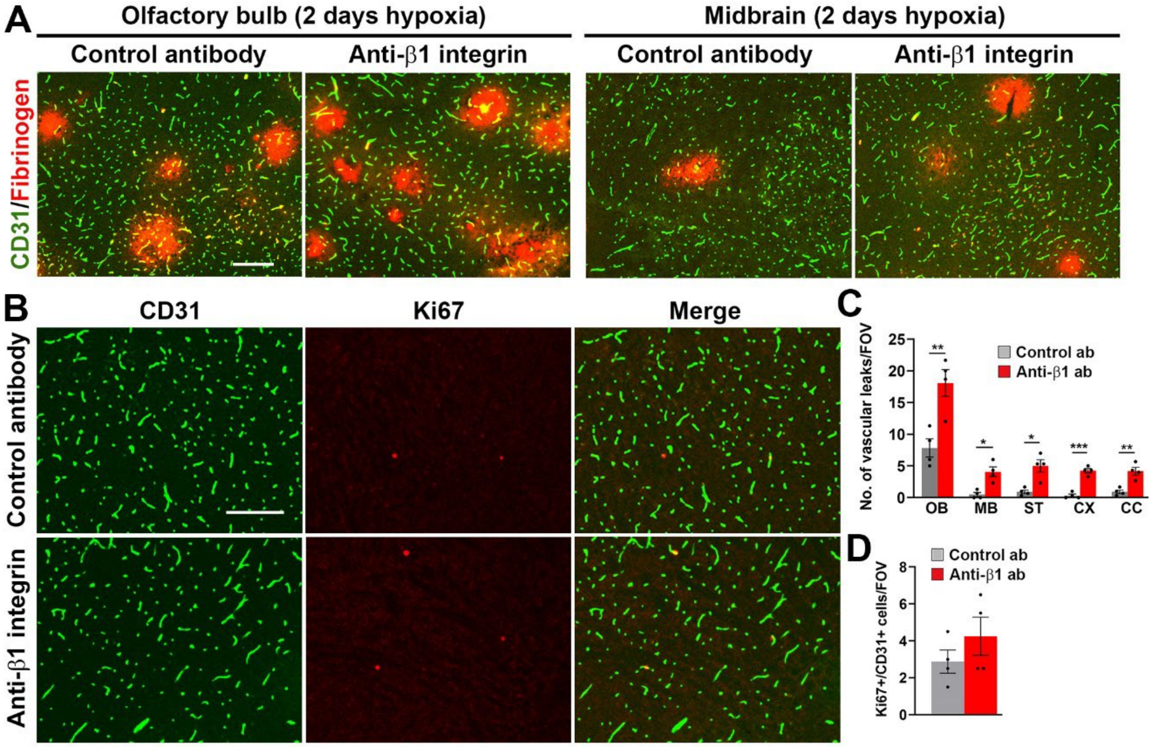

Figure S4. The impact of β1 integrin blockade on cerebrovascular leak and remodeling in aged mice after 2 days hypoxia. Frozen brain sections taken from aged (20 months) mice exposed to hypoxia (8% O_2) that received daily intraperitoneal injections of the anti-mouse β1 integrin function-blocking antibody or isotype control antibody for 2 days were stained for CD31 (AlexaFluor-488) and fibrinogen (Cy-3) **(A)** or CD31 (AlexaFluor-488) and Ki67 (Cy-3) **(B).** Scale bar = 200 µm. **C-D**. Quantification of the number of vascular leaks/FOV **(C)** or proliferating endothelial cells (CD31+/Ki67+ cells)/FOV (**D**) after 2-days hypoxia. Results are expressed as the mean ± SEM (n = 4 mice/group). * $p < 0.05$, ** $p < 0.01$, *** $p < 0.001$. Note that after 2 days hypoxia, β1 integrin blockade markedly increased the extent of hypoxia-induced vascular leak in all regions of the aged, and that at this early timepoint, only low levels of endothelial proliferation had occurred, which were not significantly affected by β1 integrin blockade. OB, olfactory bulb; MB, midbrain; ST, striatum; CX, cerebral cortex; CC, corpus callosum.

Figure S5

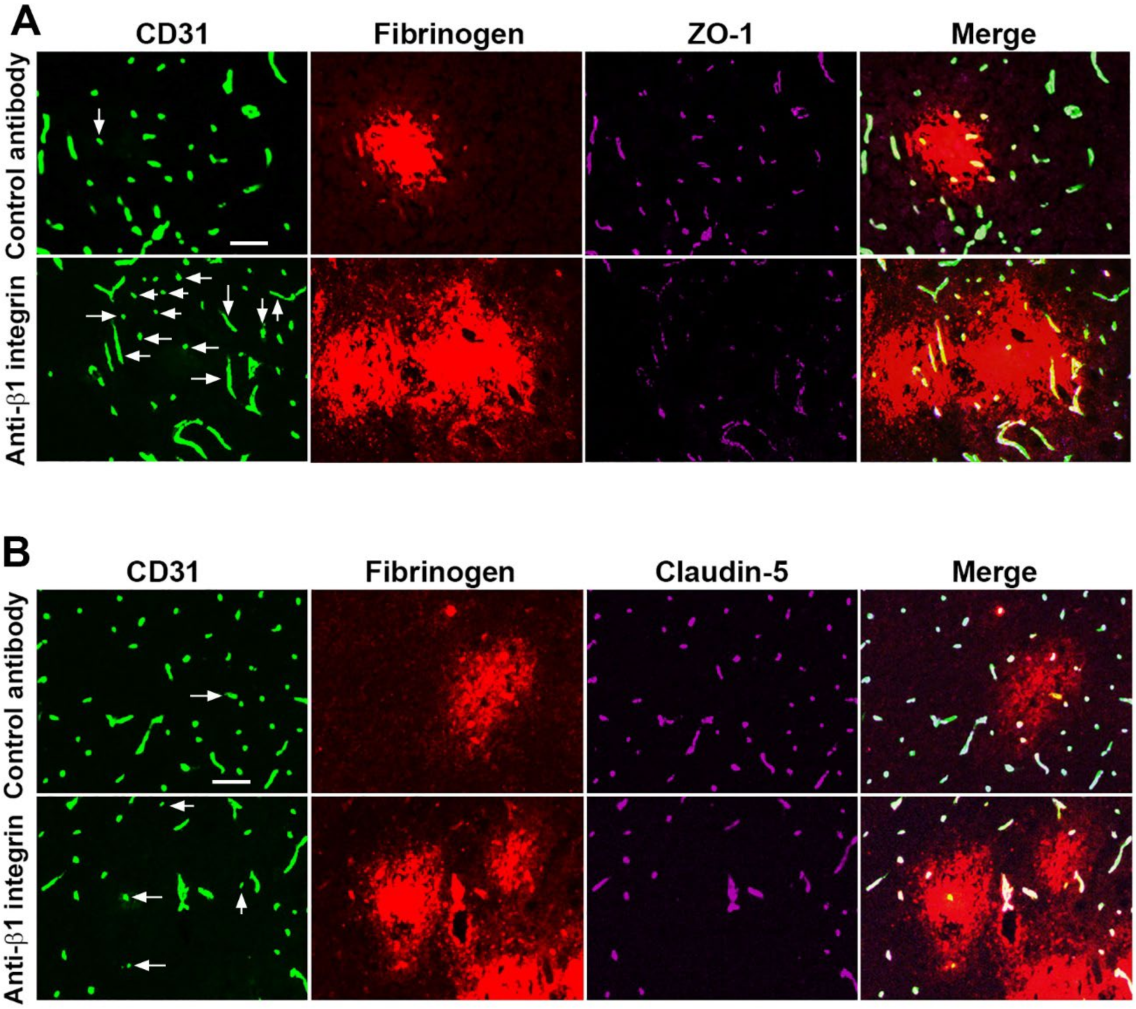

Figure S5. The impact of β1 integrin blockade on vascular tight junction protein expression. Frozen brain sections taken from young (8-10 weeks) mice exposed to hypoxia (8% O_2) that received daily intraperitoneal injections of the anti-mouse β1 integrin function-blocking antibody or isotype control antibody for 4 days were triple-stained for CD31 (AlexaFluor-488), fibrinogen (Cy-3) and ZO-1 (Cy-5) (**A**) or CD31 (AlexaFluor-488), fibrinogen (Cy-3) and claudin-5 (Cy-5) (**B**). Scale bar = 50 μm. Note that while in control mice, some blood vessels at the center of vascular leaks showed loss of tight junction proteins, the number of vessels lacking tight junction protein expression was greatly increased in mice treated with the β1 integrin function-blocking antibody (arrows).

Figure S6

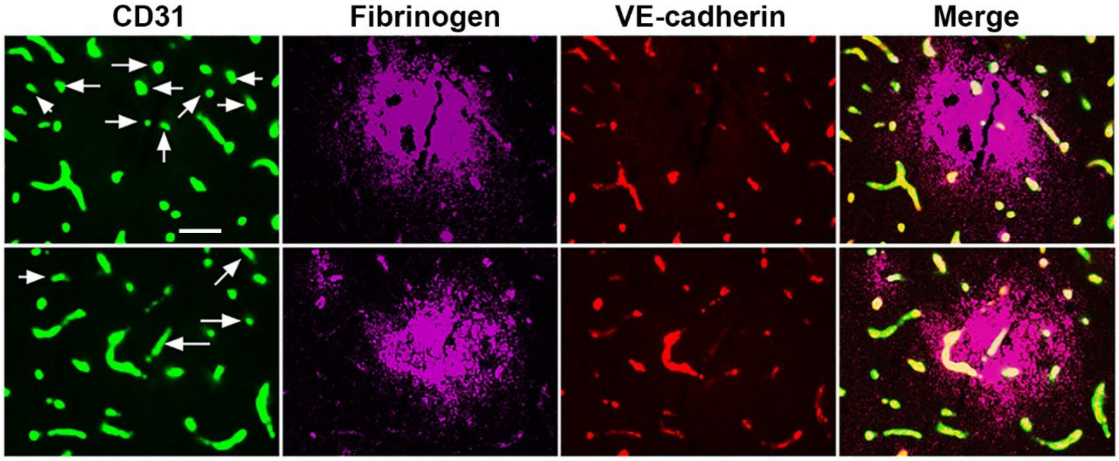

Figure S6. The impact of β1 integrin blockade on vascular VE-cadherin expression. Frozen brain sections taken from young (8-10 weeks) mice exposed to hypoxia (8% O_2) that received daily intraperitoneal injections of the anti-mouse β1 integrin function-blocking antibody for 4 days were triple-stained for CD31 (AlexaFluor-488), fibrinogen (Cy-5) and VE-cadherin (Cy-3). Scale bar = 50 µm. Note that while most blood vessels expressed strong levels of VE-cadherin, vessels at the center of vascular leaks showed greatly reduced levels of VE-cadherin (arrows).
